# Comparison of Long-Term Microscopic and Endoscopic Audiologic Results After Total Ossicular Replacement Prosthesis Surgery

**DOI:** 10.1097/MAO.0000000000003733

**Published:** 2022-11-07

**Authors:** Adrianus H.A. Baazil, Fenna A. Ebbens, Erik van Spronsen, Maarten J.F. De Wolf, Frederik G. Dikkers

**Affiliations:** Amsterdam UMC, University of Amsterdam, Department of Otorhinolaryngology, Meibergdreef 9, Amsterdam, The Netherlands

**Keywords:** Audiologic outcome, Long-term, Ossiculoplasty, Total endoscopic ear surgery, Total ossicular replacement prosthesis

## Abstract

To compare short-term and long-term outcomes after transcanal endoscope-assisted with microscope-assisted ossiculoplasty using the Fisch titanium total prosthesis (FTTP).

**Study Design:**

Retrospective chart review.

**Setting:**

Tertiary referral center.

**Patients:**

Pediatric (<18 yr of age) and adult patients undergoing ossiculoplasty with the FTTP between January 2016 and December 2019.

**Intervention:**

Transcanal endoscope-assisted (n = 30) or microscope-assisted (n = 76) ossiculoplasty with the FTTP. In the microscopic group, 48 were performed through the ear canal and 28 by retroauricular approach.

***Main Outcome* Measure:**

Short-term (3 mo) and long-term (average 20.2 mo) PTA_0.5-2kHz_ air and bone conduction thresholds were evaluated.

**Results:**

In total, 106 patients were included. Nine of 30 (30.0%) of endoscopic and 15 of 76 (19.7%) of microscopic patients were pediatric. Endoscopic preoperative air conduction PTA_0.5-2kHz_ was 52.1 ± 15.8 dB and 52.2 ± 17.9 dB for the microscopic group (*p* > 0.05). Three months postoperative endoscopic air conduction PTA_0.5-2kHz_ was 37.6 ± 17.4 dB (14.5 dB improvement) and 44.6 ± 19.9 dB (7.6 dB improvement) in the microscopic group (*p* > 0.05). Three months postoperative endoscopic PTA_0.5-2kHz_ ABG was 26.8 ± 16.6 dB and 28.4 ± 14.7 dB in the microscopic group (*p* > 0.05). Latest follow-up endoscopic air conduction PTA_0.5-2kHz_ audiogram (mean follow-up, 20.6 ± 10.4 mo) was 36.1 ± 18.2 dB (16.0 dB improvement) and 40.1 ± 16.8 dB (12.1 dB improvement) in the microscopic group (mean follow-up, 19.9 ± 10.3 mo)(*p* > 0.05). For endoscopic air conduction PTA_0.5-2kHz_, between the 3 months and latest follow-up audiogram, 25.0% showed improvement, 50.0% remained stable, and 25.0% deteriorated. In the microscopic group, 26.7% improved, 46.6% remained stable, and 26.7% deteriorated (*p* > 0.05).

**Conclusion:**

Our study shows that hearing results with the Fisch titanium total prosthesis are in line with literature. Endoscope-assisted total ossiculoplasty proves to be a suitable technique with comparable results to the microscopic approach.

## INTRODUCTION

Total ossicular chain reconstruction is often performed and an important part of otologic surgery. Despite the frequent use of endoscopes in ear surgery, only three studies with a small number of endoscopically treated patients (n < 21) with a follow-up of less than 6 months have evaluated the endoscope's role in total ossiculoplasty. Das et al. (1) described significantly better hearing results 1 month after endoscope-assisted ossiculoplasty in comparison to microscope-assisted ossiculoplasty. At 6 months after surgery both methods were shown to have comparable audiologic outcomes (endoscopic group: n = 14, follow-up 6 months, postoperative air bone gap (ABG) PTA_0.5-3kHz_ 26.2 dB). Yawn et al. (2) state that audiologic results of endoscope-assisted and microscope-assisted ossiculoplasty are equal (endoscopic group: n = 8, follow-up 6 months, postoperative ABG PTA_0.5-3kHz_ 15.9 dB). Kwinter et al. (3) found no difference between endoscope-assisted (n = 21) and microscope-assisted (n = 23) total ossiculoplasty results (postoperative air conduction PTA_0.5-4kHz_ of 29.0 vs 31.5 dB, respectively). One of the postulated advantages of endoscopic ear surgery is the wide field and high-resolution view that may benefit accurate placement of middle ear prostheses. Potential drawbacks of using an endoscope include difficulty clearing secretions due to one-handed surgery and diminished depth perception (4,5). Because only three studies have been performed evaluating short-term hearing results, more data are needed to confirm these results. Moreover, at present, no data in literature is available on the long-term audiologic results after endoscope-assisted ossiculoplasty.

In 2004, Fisch et al. (6) published their 1 year audiologic results after total microscope-assisted ossiculoplasty using the Fisch titanium total prosthesis (FTTP, Karl Storz, Tuttlingen, Germany). They reported audiologic results with a mean postoperative air conduction and ABG PTA_0.5-2kHz_ of 43.2 and 21.3 dB, respectively. Fifty-seven percent of patients had a postoperative ABG of <20 dB and 87% <30 dB. These postoperative results are similar or superior to those published in literature for other titanium and nontitanium total prostheses (7–12). More data should be presented to confirm the results of ossiculoplasty with the FTTP presented by Fisch et al. It has been stated that ABGs after ossiculoplasty deteriorate upon longer follow-up (13,14). This is explained by mechanical factors and other persistent problems such as continuing tubal dysfunction, middle ear mucosal status and/or iatrogenic scar tissue formation (6,15). Fisch et al. advise the use of a cartilage “shoe” to obtain prosthesis stability (6). It is interesting to evaluate the long-term hearing results after ossiculoplasty with the FTTP with shoe fixation.

The aim of this study is to describe the short-term and long-term hearing results after endoscope-assisted ossiculoplasty and to compare these to endoscopic results from literature. The second aim is to confirm the hearing results of microscope-assisted ossiculoplasty with the FTTP with shoe achieved by Fisch et al. In addition to air conduction and ABG, results are presented by means of the Glasgow Benefit plot. This plot incorporates the hearing of the contralateral ear and provides important information about postoperative functionality of hearing.

## MATERIALS AND METHODS

### Patients

Patients were operated between January 2016 and December 2019. They were reviewed before surgery, 3 months and 1 to 3.5 years after surgery. Of all patients, gender, age, type of surgery, operated side and indication for surgery were recorded.

### Fisch Titanium Total Prosthesis

The Fisch titanium total prosthesis (Karl Storz SE&Co. KG, Tuttlingen, Germany) is a titanium L-shaped prosthesis designed for total reconstruction of the ossicular chain. Due to its L-shape and its flexible connection (0.2 mm thin) between the shaft and the large head (5 mm diameter and 0.1 mm thin), the FTTP can be accommodated under the tympanic membrane without cartilage protection. An additional strength of the FTTP is the alterable length of its shaft. After measuring the necessary prosthesis length with a disposable depth meter, the prosthesis shaft can be precisely cut to the desired length. The 10-mm-long shaft can be cut within 0.1 mm to the desired length (6). The offset head of the FTTP enables the surgeon to visualize the oval window freely and enables accurate placement of the prosthesis shaft on the stapes footplate (6).

### Type of Surgery

Cases were divided into categories based on mastoid and canal wall status: transcanal tympanoplasty or atticotomy with intact mastoid and canal wall, canal wall up mastoidectomy (CWU), CWU with obliteration of the epitympanic and mastoid areas (CWUO), canal wall down mastoidectomy (CWD) and CWD with reconstruction of the posterior canal wall and obliteration of the mastoid cavity (CWR). In obliterated ears (both CWUO and CWR), the epitympanum and mastoid are separated from the middle ear by a midtemporal artery flap followed by the obliteration of the mastoid and epitympanum with hydroxyapatite granules (16). Microscope-assisted ossiculoplasty was performed through the ear canal in cases undergoing second/third/fourth look after CWU(O) and in those having an ossiculoplasty after CWD. In those having a CWU(O) or CWR, it was done by a retroauricular approach. For endoscopically operated cases, the total endoscopic ear surgery (TEES) classification score by Cohen et al. (17) was noted. For all cases, the prosthesis extrusion rate was evaluated. Fixation of the prosthesis in the oval window niche was achieved using various techniques (silastic shoe, cartilage shoe, cartilage wedges, no fixation). Finally, the status of the malleus and its effect on postoperative hearing results was assessed. Status of the malleus was categorized in three groups: intact, only malleus handle present and completely absent.

Microscope-assisted ossiculoplasty was performed by all three otologists (F.E., Ev.S., Md.W.). Endoscope-assisted ossiculoplasty was solely done by Md.W.

### Evaluation of Hearing Results

We calculated ABG, air and bone conduction pure-tone averages (PTAs) for 500 Hz, 1 kHz and 2 kHz (PTA_0.5-2kHz_). Audiologic outcomes 3 months after surgery and at the latest moment of follow-up (>3 months after surgery) were compared with the preoperative situation.

Overclosure was defined as bone conduction improvement >0 dB for PTA_0.5-2kHz_. Postoperative ABGs for cumulative decibel bins were evaluated. For the calculation of the PTAs and ABGs, air and bone conduction threshold levels obtained simultaneously were used. Stability of hearing was evaluated by comparing audiologic outcomes at the latest moment of follow-up audiograms to audiometric results 3 months after surgery. Results were binned in 3 categories: stable (−5 < change of air conduction between latest and 3 month audiogram <5 dB), improvement (change < −5 dB), deterioration (change >5 dB). To evaluate the functional benefit of postoperative hearing outcomes, a Glasgow Benefit Plot was made (18). This plot takes into account preoperative and postoperative air conduction levels of the (to-be-)operated ear and the contralateral ear. We defined socially acceptable hearing as a PTA_0.5-2kHz_ air conduction threshold of <35 dB.

### Statistics

t Test, χ^2^, and one-way ANOVA were performed to statistically analyze differences. *p* Values <0.05 were considered as statistically significant.

### Ethics

The authors assert that all procedures contributing to this work comply with the ethical standards of the relevant national and institutional guidelines on human experimentation and with the Helsinki Declaration of 1975, as revised in 2008. Institutional review board approval was attained (W20_313 # 20.348).

## RESULTS

Complete preoperative audiograms were available for 106 patients. Postoperative audiograms at 3 months were available for 99 of 106 (93.4%) patients, 28 of 30 (93.3%) endoscopic and 71 of 76 (93.4%) microscopic. Fifty-three (50.0% of 106) latest follow-up audiograms were present (mean time to follow-up, 20.2 ± 10.3 mo; range, 5.5–42.4 mo), 18 of 30 (60%) endoscopic and 35 of 76 (46.1%) microscopic. Mean follow-up for all endoscopic cases was 13.7 months (range, 2.5–39.7 mo) and 10.8 months (range, 1.7–42.4 mo) in the microscopic group as a whole. Mean age at surgery in the endoscopic group was 30.4 years (range, 8.2–68.4 yr) and 36.9 years (range, 6.6–75.3 yr) in the endoscopic group. Nine of 30 (30.0%) of endoscopic and 15 of 76 (19.7%) of microscopic patients were younger than 18 years. In the endoscopic group 73.3% and 67.1% of patients in the microscopic group were male. Fifty-four prostheses (50.9%) were placed in right ears and this percentage was comparable for both groups.

One hundred five procedures (99.1%) were performed for chronic otitis media with or without cholesteatoma. In one ear, conductive hearing loss was caused by a congenital absence of the stapes. In all patients, a mobile footplate was present and the stapedial suprastructure was partially or totally absent. All endoscopic cases were TEES Class 4, except for one endoscopically assisted microscopic surgery case, which was TEES Class 2. Ten of 30 (33.3%) endoscopic procedures and 22 of 76 (28.9%) microscopic procedures were revision ossiculoplasties. Extrusion of the FTTP was seen in 8 of 106 cases (7.5%). Table 1 summarizes the demographic details of our study population.

**TABLE 1:** Demographic details of a series of 106 patients undergoing ossiculoplasty with the Fisch titanium total prosthesis

Demographic Details (n = 106)	
Age (mean, range)	35.0 (6.6–75.3)
<18 yr (n)	24
≥18 yr (n)	82
Male sex, n (%)	73 (68.9%)
Right ear, n (%)	54 (50.9%)
Length of FTTP, mean (range)	4.53 (3.0–7.0)
Type of FTTP fixation	
Silastic shoe, n (%)	55 (51.9%)
Cartilage shoe, n (%)	26 (24.5%)
Cartilage wedges, n (%)	5 (4.7%)
No fixation, n (%)	7 (6.6%)
Unknown, n (%)	13 (12.3%)
Type of surgery	
Microscopic	
CWU(O), n (%)	9 (8.5%)
Second look with ossiculoplasty after CWU(O), n (%)	25 (23.6%)
Third look with ossiculoplasty after CWU(O), n (%)	5 (4.7%)
Fourth look with ossiculoplasty after CWU(O) (n, %)	2 (1.9)
Transmeatal tympanoplasty and ossiculoplasty, n (%)	13 (12.3%)
Primary ossiculoplasty in CWD, n (%)	3 (2.8%)
CWR, n (%)	19 (17.9%)
Endoscopic	
Endoscopically assisted microscopic CWU, n (%)	1 (0.9%)
Primary ossiculoplasty after CWU(O), n (%)	5 (4.7%)
Second look with ossiculoplasty after CWU(O), n (%)	15 (14.2%)
Transmeatal atticotomy with ossiculoplasty, n (%)	1 (0.9%)
Transmeatal tympanoplasty with ossiculoplasty, n (%)	4 (3.8%)
Primary ossiculoplasty in CWD and CWR, n (%)	4 (3.8%)
Primary indication for surgery	
Chronic otitis media with or without cholesteatoma, n (%)	105 (99.1%)
Congenital ossicular chain malformation	1 (0.9%
Time to follow-up in months, mean (range)	11.7 (1.7–42.4)

CWD, Canal wall down mastoidectomy; CWR, CWD with reconstruction of the posterior canal wall and obliteration of the mastoid cavity; CWU, Canal wall up mastoidectomy; CWUO, CWU with obliteration of the epitympanic and mastoid areas.

### Changes in Air Conduction

Preoperative PTA_0.5-2kH_ air conduction thresholds and postoperative changes are shown in a scattergram in Table 2. Endoscopic preoperative air conduction PTA_0.5-2kH_ was 52.1 ± 15.8 dB and 52.2 ± 17.9 dB for the microscopic group (*p* > 0.05). Three months postoperatively (mean, 3.1 ± 0.6 mo), endoscopic air conduction PTA_0.5-2kH_ was 37.6 ± 17.4 dB (14.5 dB improvement) and 44.6 ± 19.9 dB (7.6 dB improvement) in the microscopic group (*p* > 0.05). Latest follow-up endoscopic air conduction PTA_0.5-2kH_ (mean time to follow-up, 20.6 ± 10.4 mo) was 36.1 ± 18.2 dB (16.0 dB improvement) and 40.1 ± 16.8 dB (12.1 dB improvement) in the microscopic group (mean time to follow-up, 19.9 ± 10.3 mo) (*p* > 0.05).

**TABLE 2:** Scattergram of preoperative PTA_0.5-2kHz_ air conduction hearing level and the 3-mo and latest follow-up postoperative change

Preoperative PTA_0.5-2kHz_ air conduction hearing level (dB) (n = 106)										
0–10	11–20	21–30	31–40	41–50	51–60	61–70	71–80	81–90	>91	
Endoscopic (n = 30)										
0	1	1	8	5	6	4	5	0	0	
Microscopic (n = 76)										
0	0	6	16	19	15	10	6	0	4		
Postoperative change of PTA_0.5-2kHz_ air conduction level: 3 month and latest audiogram										
Worsened					No change	Improved				
≥50	40	30	20	10	0	10	20	30	40	≥50
3 mo postoperative (n = 99)										
Endoscopic (n = 28)										
0	0	0	1	3	6	7	6	3	1	1
Microscopic (n = 71)										
0	1	0	2	5	36	14	7	5	1	0
Latest audiogram (n = 53)										
Endoscopic (n = 18)										
0	0	0	1	1	5	2	4	3	1	1
Microscopic (n = 35)										
0	0	0	2	3						

### Changes in Air Bone Gap

The mean endoscopic preoperative ABG for PTA_0.5-2kHz_ was 39.7 ± 14.0 dB and 36.5 ± 14.2 dB for the microscopic group (*p* > 0.05). The endoscopic 3 month postoperative ABG for PTA_0.5-2kHz_ was 26.8 ± 16.6 dB and 28.4 ± 14.7 dB in the microscopic group (*p* > 0.05). Latest follow-up endoscopic ABG for PTA_0.5-2kHz_ was 28.1 ± 17.9 dB and 25.3 ± 17.0 dB for the microscopic group (*p* > 0.05).

For the endoscopic ABG PTA_0.5-2kHz_ 3 months after surgery, 14.3% of patients demonstrated an ABG within 0 to 10 dB, 42.9% within 0 to 20 dB and 64.3% between 0 and 30 dB. For the microscopic group, this was 12.7%, 31.0% and 60.6%, respectively (*p* > 0.05). At the latest moment of follow-up, for the endoscopic ABG PTA_0.5-2kHz_, 11.1% had an ABG within 0 to 10 dB, 55.6% between 0 and 20 dB and 61.1% within 0 to 30 dB. For the microscopic group, this was 22.9%, 45.7% and 62.9%, respectively (*p* > 0.05). Table 3 shows the distribution of postoperative ABGs. No significant differences between the endoscopic and microscopic group at 3 months after surgery were found (*p* > 0.05).

**TABLE 3:** Endoscopic vs microscopic postoperative PTA_0.5-2kHz_ air bone gap (ABG) at 3 months after surgery and at latest follow-up

Postoperative PTA_0.5-2kHz_ ABG	Postoperative 3 Months (n = 99)		Latest Audiogram (n = 53)	
	Endoscopic n (%)	Microscopic n (%)	Endoscopic n (%)	Microscopic n (%)
0–10	4 (14.3%)	9 (12.7%)	2 (11.1%)	8 (22.9%)
0–20	12 (42.9%)	22 (31.0%)	10 (55.6%)	16 (45.7%)
0–30	18 (64.3%)	43 (60.6%)	11 (61.1%)	22 (62.9%)
>30	10 (35.7%)	28 (39.4%)	7 (38.9%)	13 (37.1%)
Total	28 (100%)	71 (100%)	18 (100%)	35 (100%)

Results from endoscope-assisted and microscope-assisted ossiculoplasty are comparable (χ^2^, *p* > 0.05).

### Stability of Hearing

Forty-six of 53 patients (16 endoscopic, 30 microscopic) who had a latest follow-up audiogram also had an audiologic evaluation at 3 months after surgery. Change in air conduction was evaluated between these audiograms. For endoscopic air conduction PTA_0.5-2kHz_, 25.0% showed improvement (change >5 dB), 50.0% remained stable and 25.0% deteriorated (change < −5 dB). In the microscopic group, 26.7% improved, 46.6% remained stable and 26.7% deteriorated (*p* > 0.05). Results are summarized in Table 4.

**TABLE 4:** Endoscopic and microscopic binned air conduction change between latest follow-up and 3 months after surgery for PTA_0.5-2kHz_

Endoscopic PTA_0.5-2kHz_ Air Conduction Bins	n	%
Improvement (>5 dB)	4	25.0%
Stable (−5 < change <5 dB)	8	50.0%
Deterioration (< −5 dB)	4	25.0%
Total	16	100%
Microscopic PTA_0.5-2kHz_ air conduction bins		
Improvement (>5 dB)	8	26.7%
Stable (−5 < change <5 dB)	14	46.6%
Deterioration (< −5 dB)	8	26.7%
Total	30	100%

Endoscopic and microscopic results are equal (χ^2^, *p* > 0.05). Approximately 50% of endoscopic and microscopic hearing results at 3 months after surgery remain stable at the latest audiogram. Around 25% improve.

### Changes in Bone Conduction

The 3-month postoperative PTA_0.5-2kHz_ changes in bone conduction are shown in appendix 1, http://links.lww.com/MAO/B518. A deterioration in perception of more than 10 dB was observed in 7 of 99 patients (7.1%), of which 2 of 28 (7.1%) were endoscopic and 5 of 71 (7.0%) microscopic. In these seven patients, the drop in PTA_0.5-2kHz_ averaged 18.8 dB, showing no difference between both groups (*p* > 0.05). One case of total deafness was observed 3 years after microscopic surgery.

### Ear Status after Previous Surgery

Sixty-two individuals (58.5%) had ossiculoplasty in a closed cavity (CWU, CWUO). Forty-two of those were obliterated (CWUO). In 26 patients (24.5%), ossiculoplasty was performed after CWD or CWR. In 18 ears (17.0%), the ossicular chain was reconstructed with an intact mastoid and canal wall. No significant difference (*p* > 0.05) was found in 3 months postoperative PTA_0.5-2kHz_ ABG between CWU, CWD, CWUO, CWR, and those with an intact mastoid.

### Prosthesis Length, Fixation Method, and Status of Malleus

The average length of the prosthesis shaft for all patients was 4.53 mm (range, 3.0–7.0 mm). In CWU(O) this was 4.59 mm (range, 3.0–6.0 mm), in CWR 4.46 mm (range, 3.0–7.0 mm), in CWD 4.00 mm (range, 3.5–5.0 mm) and in patients with an intact mastoid and ear canal 4.52 mm (range, 4.0–5.50 mm). The average FTTP length was 4.72 ± 0.72 mm for endoscope-assisted and 4.45 ± 0.92 mm for microscope-assisted ossiculoplasties (*p* > 0.05). The prosthesis length had no influence on ABG improvement (*p* > 0.05).

As over 75% of patients had a shoe-fixated FTTP, this could not be compared with other fixation methods.

The 3-month postoperative ABG PTA_0.5-2kHz_ was 27.6 ± 17.1 dB for 13 cases with an intact malleus. For 60 with only the malleus handle present it was 25.6 ± 14.8 dB and in 26 with a completely absent malleus it was 33.5 ± 14.3 dB. The differences between these three groups were not statistically significant (*p* > 0.05).

### Overall Hearing Outcome

**FIG. 1:**
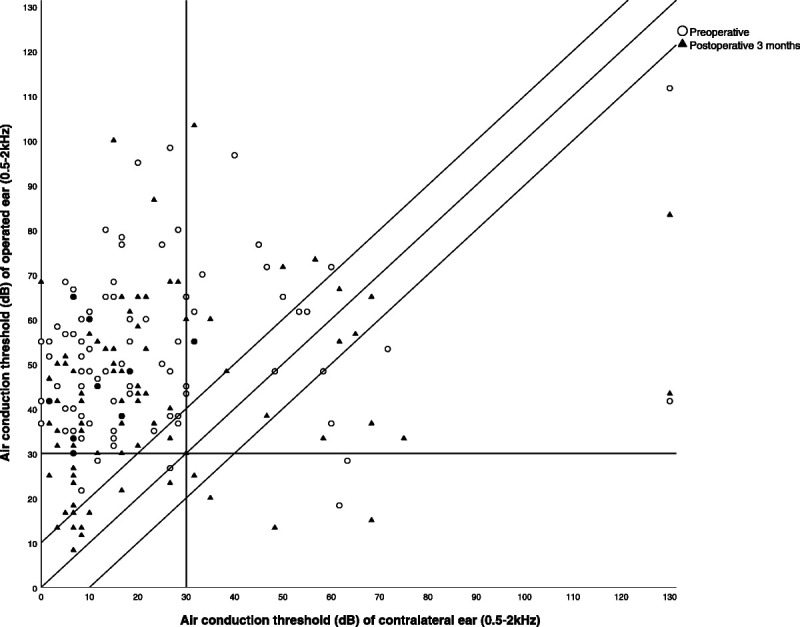
Glasgow benefit plot, 3 months after surgery (n = 99) in 24.2% of patients bilateral normal hearing and in 5.1% unilateral normal hearing (operated ear) was measured. In another 38.4% of our patients, hearing in the impaired and operated ear improved, but mean air conduction threshold levels remained above 30 dB.

Preoperative and postoperative Glasgow Benefit Plots are shown in Figure 1 and Appendix 2 (http://links.lww.com/MAO/B519). Preoperatively, 77.8% of our patients had a unilateral impairment of >30 dB on the to-be-operated ear. Three months postoperatively, 29.3% of patients had a normal hearing (air conduction threshold <30 dB) in the operated ear. Of those patients, 24.2% had bilateral normal hearing and in 5.1% unilateral normal hearing (operated ear) was achieved. In another 38.4% of patients, hearing improved after surgery, but mean air conduction threshold levels remained above 30 dB. No improvement was seen in 33.3%. A postoperative deterioration of >10 dB of the mean air conduction threshold was present in 12.1% of patients.

When the mean air conduction threshold level was set to 35 dB, 39.4% achieved socially acceptable hearing on the operated side. In 35.4% of patients bilateral and in 4.0% unilateral (operated ear), socially acceptable hearing was measured. In another 30.3% of patients, hearing in the impaired and operated ear improved, but the mean air conduction threshold levels remained above 35 dB.

## DISCUSSION

### Endoscope-Assisted vs. Microscope-Assisted Ossiculoplasty

To our knowledge, our study is the largest to primarily investigate the results of endoscope-assisted total ossiculoplasty. In addition, our follow-up is longer than previously described in literature (1–3). Das et al. (1) described significantly better hearing results 1 month after endoscope-assisted ossiculoplasty in comparison to microscope-assisted ossiculoplasty. Yet, at 6 months after surgery both methods were shown to have comparable audiologic outcomes. Yawn et al. and Kwinter et al. (2,3) state that audiologic results of endoscope-assisted and microscope-assisted ossiculoplasty are equal. Short-term endoscopic results from our study are comparable to those in literature. Our study does not confirm the postulated advantage of the superior view leading to better hearing results in endoscope-assisted ossiculoplasty as opposed to the microscope. However, this study does show that the endoscopic approach achieves comparable results to a microscopic approach. Drawbacks of endoscopic surgery, such as blood obscuring the view, one-handed surgery and diminished depth perception, do not seem to play a major role in ossiculoplasty.

### Changes in Air Conduction and Air Bone Gap

Audiologic outcomes of FTTP ossiculoplasty in our tertiary referral center are largely comparable to those published by Fisch et al. They reported a mean postoperative air conduction and ABG PTA_0.5-2kHz_ of 43.2 and 21.3 dB. Postoperative air conduction thresholds by Fisch et al. for PTA_0.5-2kHz_ do not significantly differ from our results. As they presented no standard deviations for ABG results, this could not be statistically compared to ours. Fisch et al. also describe 57% of patients having a postoperative ABG of <20 dB and 87% <30 dB, which is comparable to our results. These postoperative results are similar or superior to those published in literature for other titanium and nontitanium total prostheses (7–12). As these results are largely reproducible, the outcome of FTTP ossiculoplasty does not solely depend on surgical skills. In our opinion, the slightly lower improvement in air conduction threshold levels and ABG in comparison to Fisch et al., can be explained by a larger difference in mean preoperative air conduction threshold levels and ABGs. As we showed that conduction can improve from 3 months to latest follow-up (±20 mo), also the timing of audiologic evaluation might have contributed. In our study this was 3 months after surgery and in the study by Fisch et al. 1 year. The prostheses used in CWU(O) were longer in the study by Fisch et al. (mean, 7.9; range, 4–11 mm) when compared with our study (mean, 4.59; range, 3.0–6.0 mm). Although Fisch et al. describe better results in patients treated with longer prostheses, we found no effect of prosthesis length on outcome. As prosthesis length varied little in our study, this might possibly explain the lack of differences. In both studies, middle ear depth was measured before FTTP placement. We therefore have no explanation for the difference in closed cavity prosthesis length between Fisch et al. and our study.

In 8 of 106 cases (7.5%), the FTTP was extruded. This rate is higher than the 0% described by Fisch et al. For other TORPs described in literature, extrusion rates vary from 0% (6,19) to 16.5% (10). Others found an extrusion rate of 5% (20), 1.1% (21) and 3.8% (7). For all of these TORPs, a cartilage graft was placed between the prosthesis and the tympanic membrane. Despite no cartilage graft is necessary using the FTTP, results are comparable. As we had a longer follow-up period than Fisch et al., this might be an explanation for the difference in extrusion rate.

### Stability of Hearing Outcome

In addition to the 1-year postoperative results by Fisch et al., we described the long-term results of the FTTP. Over a mean follow-up of 2 years, for around 75% of patients hearing remained stable or improved. Twenty-five percent showed deterioration of >5 dB. We suggest that the stability of results is possibly the result of the shoe fixation of the FTTP in the oval window niche. Also, the ability to fine tune the positioning and angulation of the large flexible FTTP head in direct contact and in alignment with the tympanic membrane may have contributed to its stability.

Long-term audiologic results have been described for different nontitanium TORPs. Colletti et al. (13) demonstrated an ABG increase from 6 months to 5 years after surgery of 17.5 to 24.7 dB. Lesinski (14) also showed a tendency of decreased conduction over time (18% of TORPs ABG <25 dB 4 years after surgery). In achieving better prosthesis stability, the use of a cartilage shoe has been evaluated. In one study by Gostian et al., (22) comparing shoe-fixated with non–shoe-fixated total ossicular chain reconstruction led to a smaller ABG (17.7 vs. 21.6 dB, respectively) at the short-term (<1 yr follow-up). At longer follow-up (>1 yr), results were equal as conduction improved slightly in the non–shoe-fixated group (18.0 vs. 19.3 dB, respectively). Yung and Smith (10) evaluated the Aerial-Total-Dusseldorf total titanium prosthesis with shoe fixation (Aerial-Total-Dusseldorf, Heinz Kurz GmbH Medizintechnik, Dusslingen, Germany). He demonstrated a postoperative ABG of 20.7 dB over a follow up of 24 months (n = 28). Unfortunately, audiologic results 6 and 12 months postoperatively are not presented in the article, rendering it impossible to evaluate the long-term stability of their shoe-fixated titanium TORP. Fayad et al. (23) also evaluated their shoe-fixated long-term titanium TORP hearing outcomes. Their postoperative ABGs were comparable to those described by Yung et al. In addition to hearing outcome, this study described outcome stability comparing short-term (mean follow-up 3.4 months, n = 134) with long-term (mean follow-up, 21.7 months; n = 63) hearing results (23). Improvement was found in 23.3% and deterioration in 11.6% of cases. These results by Yung and Fayad et al. are in line with our results, but is impossible to conclude what the role of shoe fixation is in the acquired results as both studies lack a non–shoe-fixated group. In our study, the non–shoe-fixated FTTP ossciculoplasty group was too small for a reliable comparison to those with shoe fixation.

Timing of postoperative audiologic evaluation may influence the final hearing outcome. Internationally, consensus has been reached that an audiogram 3 months after surgery will result in a reliable audiogram as hearing likely has reached its final level (24). Yet, the results by Fayad et al. and our results do imply that hearing can change even after 3 months; in up to around 25% of patients hearing further improved after 3 months. This means that at 3 months after surgery, hearing outcome would be underestimated and patients should be informed of this possibility. We postulate that, although titanium has good biocompatibility (25), the middle ear mucosa has not settled completely around the titanium prosthesis at 3 months, explaining the further improvement over time. This has probably contributed to the difference in air conduction and ABGs 3 months after surgery between our study and the one by Fisch et al.

### Changes in Bone Conduction

No difference in deterioration in perceptive hearing of more than 10 dB between our study and the study by Fisch et al. was found. Three years after microscopic surgery one of our patients developed a total deafness on the operated ear. A computed tomography showed a normal piston position, and no abnormalities were found on magnetic resonance imaging, deeming a causal relation with the ossiculoplasty unlikely.

### Ear Status after Previous Surgery

No significant difference in hearing outcome was found for patients after a CWU, CWD, CWUO, and CWR. This result is in line with data in medical literature (6,26).

### Overall Hearing Outcome

Postoperative improvement of the air bone gap and air conduction levels are important when evaluating the results of ossiculoplasty. Yet these outcomes do not assess the functional benefit from improved hearing (18). With the Glasgow Benefit Plot, we demonstrated that in around 40% of cases a socially acceptable air conduction threshold of less than 35 dB on the operated side is achieved. This is an important and useful message for patient counseling as it means that they might no longer need a hearing aid after surgery.

## CONCLUSION

Our study shows that hearing results with the Fisch titanium total prosthesis are in line with literature. Endoscope-assisted total ossiculoplasty proves to be a suitable technique with comparable results to the microscopic approach.

## Supplementary Material

SUPPLEMENTARY MATERIAL
